# Dietary branched-chain amino acid assessment in broilers from 22 to 35 days of age

**DOI:** 10.1186/s40104-020-00535-1

**Published:** 2021-01-10

**Authors:** M. T. Kidd, F. Poernama, T. Wibowo, C. W. Maynard, S. Y. Liu

**Affiliations:** 1 Center of Excellence for Poultry Science, Division of Agriculture, University of Arkansas System, Fayetteville, AR 72701 USA; 2 PT JAPFA Comfeed Indonesia Tbk, Jakarta, 12810 Indonesia; 3 School of Life and Environmental Sciences, Faculty of Science, The University of Sydney, Brownlow Hill, Sydney, NSW 2570 Australia

**Keywords:** Broiler, Crude protein, Isoleucine, Leucine, Response surface, Valine

## Abstract

**Background:**

Valine and isoleucine are similar in chemical structure and their limitation in broiler chicken diets. To evaluate their limitation and interactive effects, multivariate assessment nutrition studies for the branched-chain amino acids (BCAA) are needed. A three level (− 1, 0, + 1), three-factor Box-Behnken design study was conducted to assess dietary BCAA ratios to lysine of 65, 75, and 85 for valine, 58, 66, and 74 for isoleucine, and 110, 130, and 150 for leucine in male and female Lohman Indian River broilers from 22 to 35 d of age.

**Results:**

Live performance of male broilers was not affected by BCAA level. However, male broilers fed increasing isoleucine had improved (*P* = 0.07) carcass yield as leucine and valine were reduced. Female broilers had improved body weight gain (*P* = 0.05) and feed conversion (*P* = 0.003) when leucine and isoleucine were at their lowest levels, independent of valine, but increasing leucine impaired live performance and warranted concomitant increases in isoleucine to restore responses. Increasing dietary isoleucine and valine in female broilers increased breast meat yield (*P* = 0.05), but increasing leucine tended to diminish the response.

**Conclusion:**

The female Lohman Indian River broiler is more sensitive to BCAA diet manipulation than males. Specifically, as dietary leucine is increased in female broilers, dietary isoleucine increases were needed to offset the negative effects. Both increases in dietary valine and isoleucine improved breast meat yield in female broilers, but only when birds were fed the lowest dietary leucine.

## Background

The similarity among the branched-chain amino acid (BCAA) side chains has resulted in broiler chicken nutritional studies conducted to assess them in concert for over half a century. Although research results on interactive, synergetic, and antagonistic effects of BCAA vary, the negative impact of high dietary leucine on broiler performance does not [1]. Farron et al. [2] conducted a study to assess interactions between dietary BCAA on metabolic function and performance in broilers. Excess leucine in broilers fed low crude protein (CP) diets did not result in metabolic ketosis, but excess leucine did decrease overall bird performance [2]. Further, as more feed grade amino acids enter feed formulation with attractive pricing, diets fed to broilers will continue to have less CP. Therefore, continued assessment of BCAA in practical broiler diets is warranted.

Broiler diets formulated solely on a vegetable basis or that with meat meal inclusions typically result in valine and isoleucine, respectively, positioned as the fourth limiting amino acid in the nutrient matrix [3]. The continued reduction in CP requires a higher dietary inclusion of feed grade amino acids, which may alter digestion and absorption rates. Because both L-valine and L-isoleucine have become available to poultry nutritionists in feed grade form, and the BCAA have similar digestion and absorption patterns, research is needed to assess concurrent BCAA responsiveness in modern broilers fed on practical diets. Farren and Thomas [4] demonstrated that the BCAA can be simultaneously decreased without decreasing broiler performance, but an individual decrease in valine decreases broiler performance.

Due to the synergies in BCAA chemistry and their limitation in low CP diets for broilers, the work employed made use of the Box-Behnken experimental design. The objective of the current study was to assess male and female broiler responsiveness to dietary valine, isoleucine, and leucine ratios to lysine via a three-factor Box-Behnken response surface experimental design.

## Materials and methods

### Bird husbandry and management

Lohmann Indian River chicks sourced from a commercial hatchery and feather sexed were used for sex-separate experiments. Prior to incubation, all eggs collected from broiler breeders were of a similar age. Male (4,500 chicks) and female (4,800 chicks) broilers were placed in floor pens in a closed sided house. Each experiment utilized 130 floor pens measuring 1.44 m × 1.70 m and was conducted from 22 to 35 d of age. At d 22, both male and female bird numbers were adjusted to 34 (13.9 birds per m^2^) and 36 (14.7 birds per m^2^), respectively.

The closed sided wall facility used radiant heating during brooding, negative pressure ventilation for air exchange during brooding, and tunnel ventilation for cooling during the 22 to 35 d experimental periods. Each pen was equipped with one tube feeder, one nipple drinker line, and new rice hulls for bedding. The light procedure followed the primary breeder recommendation for Lohmann Indian River broilers [5]. Feed and water were provided ad libitum. Mortality were obtained daily and their weights were recorded to calculate adjusted feed conversion by adding mortality weight to pen body weight at day 35. Bird care followed research protocol and ethics guidelines established by the JAPFA Animal Care Committee. Any mortality exceeding the primary breeder recommendation required flock oversight from a veterinarian.

### Experimental design and diets

Both male and female chicks were offered a proprietary common starter feed in crumbled form from 1 to 21 d of age. A basic test diet primarily composed of corn, soybean meal, and peanut meal was formulated to create 13 treatment variations in BCAA that were administered from 22 to 35 d of age and shown in Table 1. In both male and female experiments, varying levels of L-valine, L-isoleucine, and L-leucine were used to create the 3-factor treatments of the Box-Behnken experimental design. Two fillers were used (i.e., palm olein and washed sand) so that the 13 BCAA treatments would be equal in energy and diet total volume. The 13 BCAA treatments were expressed on a fecal digestible basis relative to dietary lysine. Each treatment was replicated 10 times in each experiment. All treatment diets were pelleted after mixing with a conditioning temperature range from 78 to 80 ℃. Composite samples of pelleted dietary treatments were obtained, ground, hydrolyzed in acid, and analyzed for amino acids [6].

**Table 1:** Basic test diet formulated to allow for 13 treatment combinations^a^ varying in valine, isoleucine, and leucine for the response surface experiments in 22 to 35 days old male and female broilers

Items	Inclusion, %
Corn	63.90
Peanut meal	22.00
Soybean meal	3.74
Palm olein	2.30
Palm olein filler^a^	2.00
Limestone	1.62
Mono-calcium phosphate	0.68
L-Lysine HCl	0.84
DL-Methionine	0.43
L-Threonine	0.34
L-Valine	0.34
L-Isoleucine	0.33
L-Tryptophan	0.07
Sodium bicarbonate	0.40
Vitamin and phytase premix^b^	0.25
Sodium chloride	0.18
Potassium carbonate	0.16
Mycotoxin binder	0.15
Choline chloride	0.13
Washed sand filler^a^	0.08
Mineral premix^c^	0.06
Total	100.0
Calculated nutrients, % unless otherwise noted	
Metabolizable energy, kcal/kg	3100
Lysine, digestible %	1.13
Methionine + cysteine, digestible %	0.85
Threonine, digestible %	0.76
Tryptophan, digestible %	0.20
Arginine, digestible %	1.25
Valine, digestible %	0.74
Isoleucine, digestible %	0.66
Leucine, digestible %	1.24
Calcium, total %	0.80
Non-phytate phosphorus, total %	0.23
Sodium, total %	0.18

^a^The 13 experimental treatments were created by varying levels of L-valine, L-isoleucine, and L-leucine to achieve BCAA digestible ratios to lysine (Table 2). Palm olein and washed sand fillers were provided in varying amounts to assure all 13 treatment diets were equal in space and energy. The test diet was formulated to the lowest level of leucine from intact ingredients, and the highest levels of valine and isoleucine using a mixture of intact ingredients and feed grade amino acids

^b^Vitamin premix contained per kg of diet: vitamin A, 9030 IU; vitamin D_3_, 2580 IU; vitamin E, 21.50 IU; vitamin K_3_, 3.01; cobalamin, 0.017 mg; riboflavin, 7.74 mg; D-Pantothenic Acid, 12.04 mg; niacin, 38.7 mg; folic acid, 1.08 mg; pyridoxine, 3.01 mg; thiamine, 2.15 mg; biotin, 0.052 mg; and phytase provided 750 FTU/kg diet

^c^Mineral premix provided per kg of diet: Mn, 65 mg; Zn, 58 mg; Co, 0.14 mg; Cu, 4 mg; I, 0.22 mg; Se, 0.11 mg

Measurements for male and female experiments followed identical procedures. Pen body weight was obtained at 22 and 35 d and feed intake was measured from the 22 to 35 d period. Mortality was measured daily. Feed conversion (as FCR) was calculated for the 22 to 35 d period and represented total pen feed intake divided by total pen body weight gain. In each experiment at d 35, two birds per pen were randomly selected, weighed, and processed manually (260 birds total or 20 birds per treatment in each experiment). Once dressed carcasses were obtained, abdominal fat was removed and breast meat was manually excised from hot carcasses. Processing measurements consisted of carcass yield, and abdominal fat percentage, and total breast yield relative to body weight.

### Statistical analysis

Pen was the experimental unit for all analyses. The Box-Behnken design has been shown to be an effective multivariate design to create dietary amino acid surface plots while requiring less experimental units (i.e., pens) than a complete factorial analyses [7], which was employed to assess the BCAA concurrently. The three level (− 1, 0, + 1) three-factor Box-Behnken design was analyzed using the PROC RSREG procedure [8] for linear, quadratic, and interactive effects. Response surface plots were constructed for parameters analyzed with *P* < 0.10.

## Results

The test diet used to construct BCAA levels for response surface treatments is presented in Table 1. Digestible nutrients are presented in Table 1. Analyzed amino acid treatment additions agreed with calculated treatment levels (Table 2). Predicted means of live performance and dressed yields are presented in Table 3 and their respective *P* values are presented in Table 4. Mortality averaged 0.98% for female broilers and 1.31% for male broilers, and treatment differences did not occur.

**Table 2:** Branched-chain amino acid (BCAA) calculated treatments and subsequent analyzed CP and amino acids in the experimental diets

BCAA/Lys dietary treatments			Dietary analysis				
Val	Ile	Leu	CP	Lys	Val	Ile	Leu
65	58	130	18.64	1.20	0.86	0.72	1.59
65	66	110	18.80	1.20	0.82	0.79	1.37
65	66	150	19.28	1.22	0.83	0.79	1.78
65	74	130	18.31	1.13	0.79	0.83	1.56
75	58	110	18.63	1.17	0.91	0.70	1.38
75	58	150	18.20	1.17	0.92	0.70	1.77
75	66	130	19.13	1.20	0.92	0.78	1.58
75	74	110	19.02	1.21	0.94	0.87	1.40
75	74	150	19.46	1.22	0.95	0.89	1.81
85	58	130	18.13	1.20	1.03	0.70	1.59
85	66	110	18.45	1.19	1.04	0.79	1.41
85	66	150	18.88	1.21	1.05	0.80	1.80
85	74	130	18.51	1.22	1.04	0.86	1.59

**Table 3:** Predicted means for live performance and dressed yields as affected by variation of diet branched-chain amino acids^a^ fed from 22 to 35 d to female and male Indian River broilers^b^

Treatment			Live performance^c^			Dressed yields^d^		
Val	Ile	Leu	BW gain, kg/bird	Feed:Gain, kg:kg	Mortality, %	Carcass, %	Breast, %	Fat, %
Female broilers								
65	58	130	1.122	1.92	0.17	69.71	25.93	2.11
65	66	110	1.107	1.95	1.18	69.16	26.28	1.94
65	66	150	1.061	1.96	0.97	69.74	26.46	2.00
65	74	130	1.124	1.94	1.56	70.09	26.96	1.97
75	58	110	1.163	1.88	0.31	69.97	26.15	1.92
75	58	150	1.072	2.00	1.08	69.35	25.88	2.00
75	66	130	1.088	1.94	0.83	70.64	26.25	1.85
75	74	110	1.107	1.96	1.98	69.92	26.66	1.93
75	74	150	1.133	1.92	1.08	69.99	26.18	1.88
85	58	130	1.121	1.93	0.66	69.79	26.46	1.90
85	66	110	1.094	1.91	0.97	70.01	26.74	1.86
85	66	150	1.074	1.97	1.04	68.89	25.82	1.83
85	74	130	1.124	1.91	0.94	70.00	26.24	1.94
SE^e^ center point			0.0289	0.026	0.509	0.439	0.321	0.099
SE edge points			0.0251	0.023	0.441	0.381	0.278	0.085
Male broilers								
65	58	130	1.382	1.89	1.36	69.83	25.68	1.73
65	66	110	1.394	1.85	0.92	69.80	26.23	1.74
65	66	150	1.337	1.89	1.14	70.01	26.17	1.67
65	74	130	1.379	1.81	−0.48	70.16	26.48	1.61
75	58	110	1.419	1.86	1.84	70.26	25.78	1.74
75	58	150	1.349	1.98	2.79	69.41	25.47	1.69
75	66	130	1.342	1.92	1.47	69.87	25.71	1.86
75	74	110	1.381	1.86	1.03	69.77	26.23	1.53
75	74	150	1.403	1.86	1.69	70.27	26.31	1.63
85	58	130	1.388	1.89	1.07	69.95	25.35	1.72
85	66	110	1.378	1.84	0.92	70.16	25.80	1.63
85	66	150	1.386	1.91	2.32	69.59	25.62	1.74
85	74	130	1.407	1.84	0.99	69.98	25.83	1.57
SE^e^ center point			0.0402	0.034	0.598	0.375	0.293	0.081
SE edge points			0.0348	0.029	0.517	0.325	0.254	0.071

^a^Branched-chain amino acids represent: dietary digestible levels of valine, isoleucine, and leucine in ratio to dietary digestible lysine

^b^Coding coefficients for branched-chain amino acids of −, 0, and + were used and arithmetic means were used to estimate predicted means for linear, quadratic, and interaction regression analyses using the RSREG procedure of SAS. Each value represents the predicted mean of 10 replicates (36 birds per replicate). The average body weight for female and male broilers was 1.107 and 1.380, respectively, for both the arithmetic and predicted means

^c^Live performance represents: body weight (BW) gain in kg per bird from the 15 to 35 d period; feed:gain represents kg feed intake divided by body weight gain for the 15 to 35 d period without the mass of birds that died; and mortality represents percentage birds that died from the 15 to 35 d period

^d^Dressed yields represents: hot carcass, total breast muscles, and abdominal fat relative to live BW of processed birds

^e^SE represents: standard errors of center point means (valine, isoleucine, and leucine of 75, 66, and 130, respectively) and standard errors of edge point means (additional 12 treatments)

**Table 4:** *P* values for broiler live performance and dressed yields as affected by variation of diet branched-chain amino acids from 22 to 35 d fed to female and male Indian River broilers

	Live performance^b^			Dressed yields^c^		
*P* values^a^	BW gain, kg/bird	Feed:Gain, kg/kg	Mortality, %	Carcass, %	Breast, %	Fat, %
Female broilers						
*Linear*						
Val	0.894	0.924	0.685	0.023	0.503	0.317
Ile	0.031	0.156	0.449	0.638	0.504	0.224
Leu	0.386	0.786	0.958	0.029	0.358	0.706
*Quadratic*						
Val × Val	0.996	0.888	0.918	0.061	0.550	0.400
Ile × Ile	0.074	0.697	0.918	0.512	0.929	0.224
Leu × Leu	0.842	0.408	0.472	0.029	0.792	0.924
*Interaction*						
Ile × Val	0.990	0.459	0.277	0.848	0.053	0.371
Leu × Val	0.648	0.340	0.786	0.055	0.088	0.660
Leu × Ile	0.045	0.003	0.104	0.430	0.749	0.473
Male broilers						
*Linear*						
Val	0.392	0.268	0.541	0.753	0.897	0.314
Ile	0.156	0.309	0.836	0.540	0.842	0.067
Leu	0.206	0.406	0.229	0.747	0.322	0.587
*Quadratic*						
Val × Val	0.537	0.112	0.116	0.878	0.727	0.157
Ile × Ile	0.254	0.367	0.781	0.764	0.757	0.023
Leu × Leu	0.559	0.742	0.229	0.959	0.359	0.119
*Interaction*						
Ile × Val	0.789	0.617	0.142	0.681	0.584	0.812
Leu × Val	0.426	0.645	0.327	0.295	0.856	0.264
Leu × Ile	0.256	0.113	0.806	0.073	0.505	0.352

^a^Linear, quadratic, and interaction regression analyses parameters for predicted means were generated using the RSREG procedure of SAS. All parameters have a *d*_f_ of 1

^b^Live performance represents: body weight gain in kg per bird from the 15 to 35 d period; feed:gain represents kg feed intake divided by BW gain for the 15 to 35 d period without the mass of birds that died; and mortality represents percentage birds that died from the 15 to 35 d period

^c^Dressed yields represents: hot carcass, total breast muscles, and abdominal fat relative to live body weight of processed birds

**Fig. 1:**
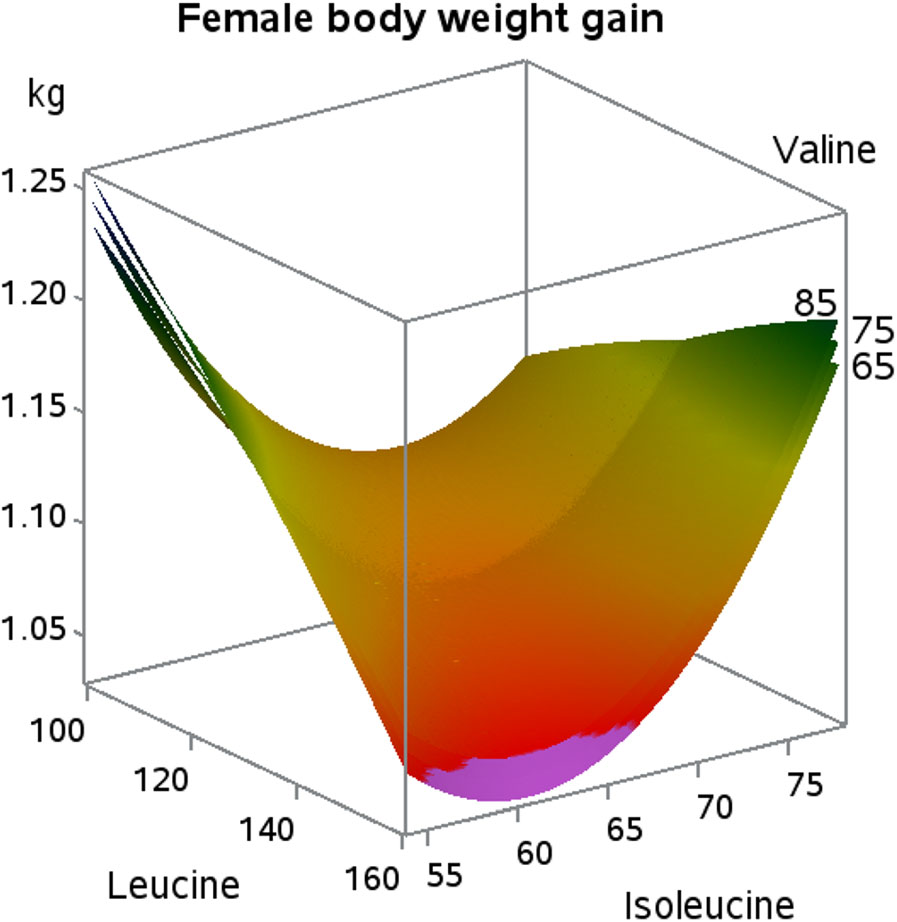
Response surface plot displaying body weight gain (kg/bird) response (*P* = 0.05; Leu × Ile interaction) at three levels of valine in female Indian River broilers

For body weight gain, quadratic responses to isoleucine (*P* = 0.07) were observed in female broilers, but no effects were observed in male broilers. An interaction occurred for body weight gain in female broilers (*P* = 0.05) where isoleucine improved body weight gain across dietary leucine levels, but the amount of dietary isoleucine needed to improve body weight gain increased with increasing leucine (Fig. 1).

**Fig. 2:**
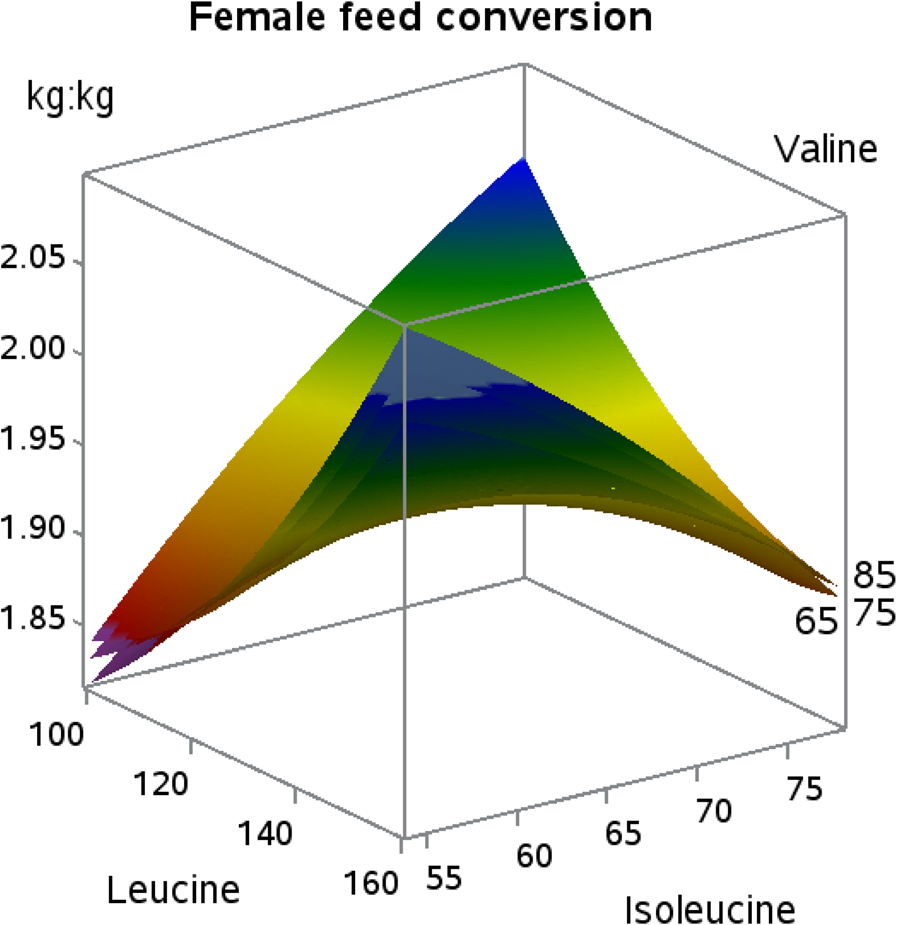
Response surface plot displaying feed conversion (kg/kg) response (*P* > 0.01; Leu × Ile interaction) at three levels of valine in female Indian River broilers

Quadratic responses for feed conversion did not occur in female or male broilers, but an isoleucine by leucine interaction (*P* < 0.01) occurred in female broilers as shown in Fig. 2. Isoleucine compromised feed conversion when dietary leucine was low, but improved feed conversion when leucine was increased.

**Fig. 3:**
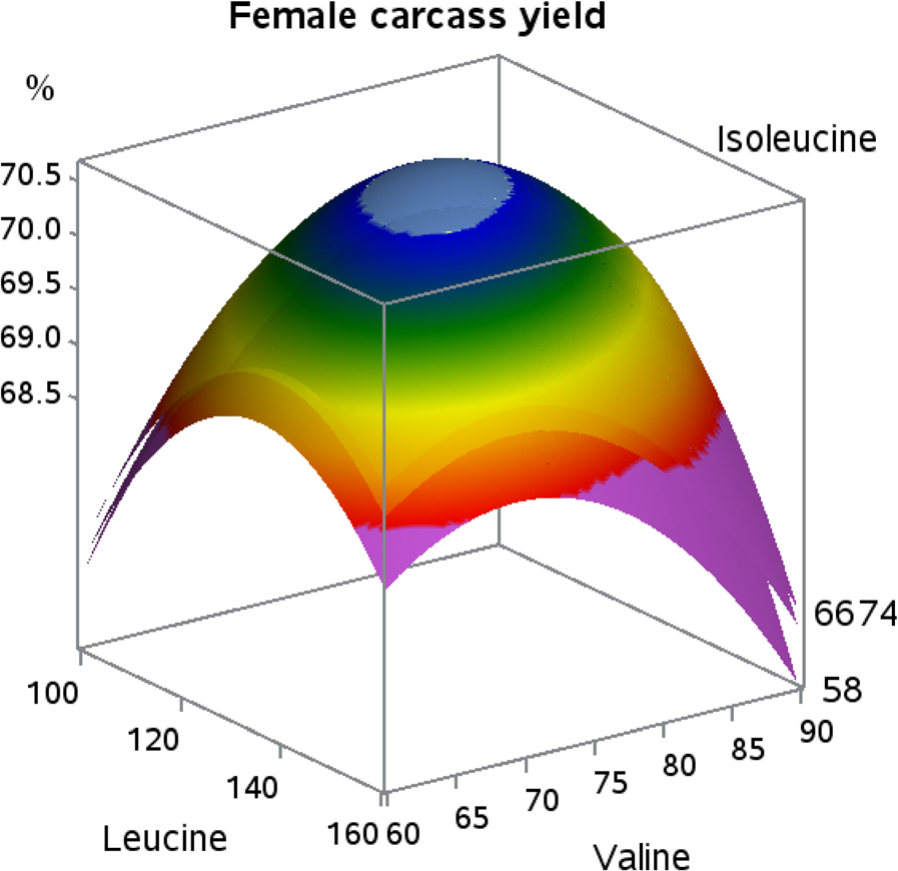
Response surface plot displaying carcass yield relative to body weight (*P* = 0.06; Leu × Val interaction) at three levels of isoleucine in female Indian River broilers

**Fig. 4:**
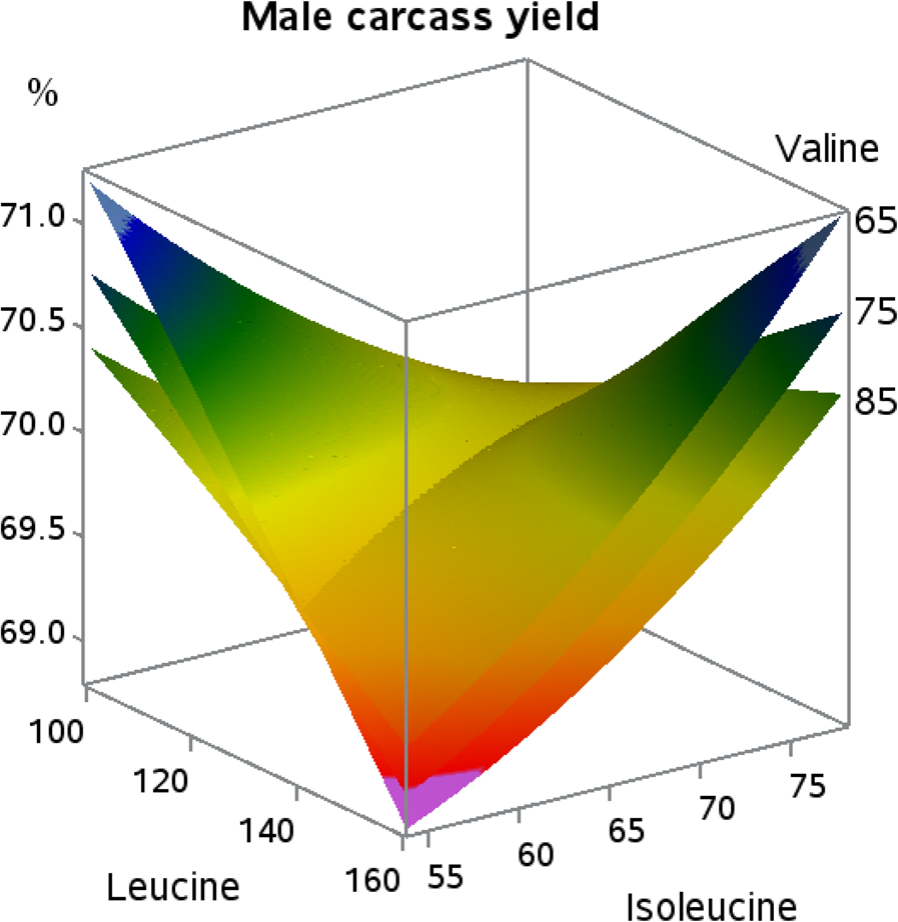
Response surface plot displaying carcass yield relative to body weight (*P* = 0.07; Leu × Ile interaction) at three levels of valine in male Indian River broilers

Dietary valine and leucine responses in female broilers resulted in quadratic responses for carcass yield. Linear or quadratic responses for carcass yield in male broilers to dietary BCAA did not occur. The interaction (Fig. 3) indicated that as dietary leucine reached 129 and valine reached 75, carcass yield in female broilers was heightened (*P* = 0.06). The only interaction for male broilers was in carcass yield as shown in Fig. 4. Male carcass yield was improved with either the combination of the lowest levels of dietary leucine and isoleucine, or the highest levels of leucine and isoleucine, with high dietary leucine in the presence of low isoleucine resulting in the poorest carcass yield response (*P* = 0.07).

**Fig. 5:**
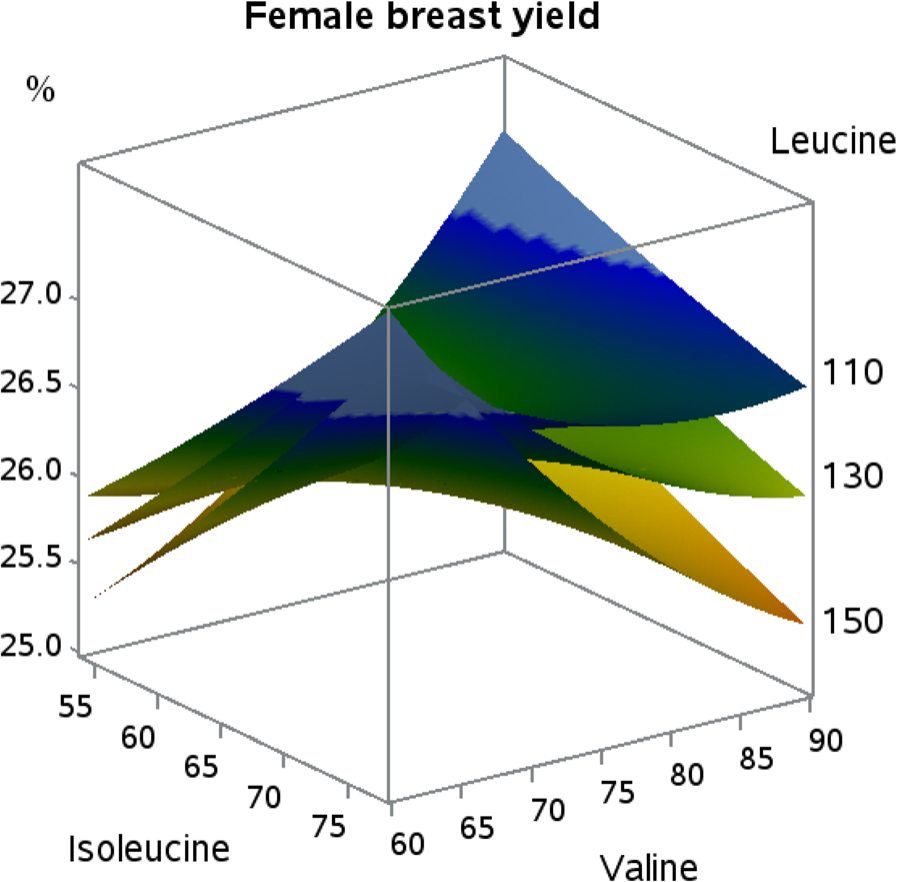
Response surface plot displaying total breast meat yield relative to body weight (*P* = 0.05; Ile × Val interaction) to isoleucine and valine at three levels of leucine (*P* = 0.09; Leu × Val interaction) in female Indian River broilers

Male breast meat yield responses were not affected by the dietary BCAA, but isoleucine by valine (*P* = 0.05) and leucine by valine (*P* = 0.09) interactions in female broilers are shown in Fig. 5. Female broilers fed the lowest levels of isoleucine and leucine in the presence of the highest dietary valine have the best breast meat yield responses. However, the opposite response occurred at the lowest level of valine where both isoleucine and leucine were needed to improve breast meat yield in female broilers.

Female dietary BCAA responses for abdominal fat yield did not occur, but male broilers had linear and quadratic responses to dietary isoleucine (*P* = 0.07 and *P* = 0.02, respectively). The female isoleucine response indicates reduced fat yields when dietary levels are increased above a ratio of 66 to lysine.

## Discussion

The highest body weight gain was achieved when dietary isoleucine and leucine levels were at their lowest values, followed by isoleucine restoring body weight from the negative effect of increasing leucine, regardless of dietary valine concentration. Recent data in males found *an interaction* between isoleucine and valine in Cobb 500 broilers that indicated that valine was the key BCAA influencing body weight gain when leucine levels fluctuated [9]. The feed conversion responses in the current study followed a similar trend where feed conversion values were lowest when isoleucine and leucine values were at their lowest or highest dietary levels, independent of valine. This response varied from similar work in Cobb broilers [9] that demonstrated a dietary imbalance between isoleucine and valine optimized feed conversion. During the experimental period used in the current work (22 to 35 d of age), female broiler breast meat accretion is occurring at a more rapid rate than that of males. This is evident by the fact that both males and females have similar breast meat yields early in life, but female breast meat yield eclipses that of males at d 35 [10]. Increasing dietary leucine beyond estimated requirement levels has been shown to decrease breast meat yield and quadratic responses in body weight gain have been observed to dietary leucine [11, 12]. Although leucine can independently create negative effects in broilers, antagonistic effects among the BCAA have been known since 1968 [13]. The root cause of this antagonism is attributed to leucine levels, and an increase in any of the three can be overcome by increasing the dietary level of the other two [14, 15]. The offset points where optimal body weight gain and feed conversion are located indicate that these amino acids must be present in balance to prevent antagonism, with emphasis on lower isoleucine and leucine inclusion, produce an optimal dietary BCAA profile for live performance measurements. Further, it must be pointed out that the success of this central composite design relied upon diet addition of BCAA L-form amino acids, which have been shown to be digested fasted than their protein-bound forms [16], due to their hydrophobic chemical nature. In addition to digestion, BCAA rely on the large neutral amino acid transporter protein 1 [17] for absorption. Hence, it may be that BCAA balance is also related to large neutral amino acid balance in diet formulation for poultry.

Female carcass yield responses indicate that optimal leucine and valine to lysine ratios are around 130 and 75, respectively, due to the presence of an apex. It is interesting that the interaction between isoleucine and leucine observed in body weight gain was not carried over to carcass yield, but instead transitioned to an interaction between isoleucine and valine. Isoleucine and valine have not been shown to influence carcass yields of female broilers when tested individually in requirement studies, again pointing to interconnectivity of the BCAA [18–20]. The negative effect of leucine on breast meat yield was expected as previously discussed with live performance. The overall response surface for female breast meat yield is similar to that of male broilers reported in previous work [21], with the exception that those researchers indicated that valine was the key BCAA in breast meat yield. Further, increasing valine levels resulted in a decrease in breast meat yield, whereas changes in isoleucine levels resulted in relatively stable breast meat yields [21]. In the current work, increasing dietary isoleucine levels resulted in increased breast meat yield, agreeing with the findings of Hale et al. [18] who utilized similar genetics to the work herein. The extent to which isoleucine and valine affect breast meat yield appears to vary depending on the level of leucine, likely due to the detrimental effects of BCAA imbalance exacerbated by the increasing amounts of leucine, and their inverse relationship is likely due to different metabolic functions. While increases in isoleucine result in increased white meat yield, increases in dietary valine have been shown to increase dark meat yield [18, 22]. Compounding these responses, the proportion of white meat to dark meat would incrementally change through differences in the parts yields that make up those skeletal muscle components, appearing in the current study as a significant response in breast meat yield.

## Conclusion

The present study demonstrates the sensitivity of the Lohman Indian River female broiler to leucine on live performance measures. Hence, in females fed low dietary leucine, and isoleucine at the lowest level, resulted in good performance (i.e., body weight gain and feed conversion), but as leucine increased in the diet, good performance was dependent on concomitant increases in dietary isoleucine. Further improvements in females were noted with breast meat yield to increasing dietary isoleucine, pending low dietary valine. However, dietary valine independently improved female breast meat yield when both leucine and isoleucine were at their lowest levels. Dietary formulation software should monitor leucine levels, and future isoleucine and valine needs of Indian River Broilers should be assessed in diets formulated to minimized leucine.

## Data Availability

Data may be provided following request to the corresponding author.

## Abbreviations

BCAA: Branched-chain amino acids
CP: Crude protein
ME: Metabolizable energy
